# Reply to ‘Fetal side’ of the placenta: Anatomical mis-annotation of carbon particle ‘transfer’ across the human placenta

**DOI:** 10.1038/s41467-021-26438-x

**Published:** 2021-12-03

**Authors:** Eva Bongaerts, Hannelore Bové, Ivo Lambrichts, Nelly D. Saenen, Wilfried Gyselaers, Michelle Plusquin, Maarten B. J. Roeffaers, Marcel Ameloot, Tim S. Nawrot

**Affiliations:** 1 Centre for Environmental Sciences, Hasselt University, Agoralaan Building D, 3590 Diepenbeek, Belgium; 2 Biomedical Research Institute, Hasselt University, Agoralaan Building C, 3590 Diepenbeek, Belgium; 3 Centre for Surface Chemistry and Catalysis, KU Leuven, Celestijnenlaan 200F-Box 2461, 3001 Leuven, Belgium; 4 Department of Obstetrics, East-Limburg Hospital, Schiepse Bos 6, 3600 Genk, Belgium; 5 Department of Public Health and Primary Care, KU Leuven, Herestraat 49-Box 706, 3000 Leuven, Belgium

**Keywords:** Cell biology, Anatomy

**replying** **to** B. Holder et al. *Nature Communications* 10.1038/s41467-021-26437-y (2021)

The Matters Arising article from Holder et al. argues that the finding of carbon particles reaching the fetal side of the human placenta is based on anatomical misinterpretation and that we identified its presence mainly in the trophoblast layer of the placental villous tissue. Instead, Holder et al. presume the location of carbon particles being in the placental villous tissue, more specifically, the trophoblast layer. Their concerns are primarily based on the use of the term “fetal side of the placenta”, which can be defined as (i) the sample collection site of the fetal biopsy, namely at the placental side facing the fetus or (ii) the anatomical site that is in direct contact with the fetal circulation (i.e., fetal capillaries). Whereas we used the terms “fetal and maternal side” of the placenta to define our biopsies as done in several previous studies^1–5^, we agree that there is no unambiguous boundary between the fetal and maternal side of the placenta being separated by the intervillous space and that more conventional terms (e.g., the basal and chorionic plate, respectively) should be used for future reference to avoid possible confusion. Although full macro- and microscopic analysis of the placental structure and particle localization was outside the scope of our paper, which aimed to look for the presence of carbonaceous particles from ambient exposure in the placenta per se, we have further evidence for the presence of carbon particles in fetal microvessels (vide infra). Hence, our claim that carbon particles reach the fetal side of the human placenta is valid irrespective of the used definition for the term “fetal side of the placenta”.

We first wish to further clarify how we carried out the two types of biopsies. As referred to in our “Methods” section, a detailed description of the placental biopsy sampling is provided in the ENVIRonmental influence *ON* AGEing in early life (ENVIR*ON*AGE) birth cohort profile^6^. The fetal-sided biopsies were collected from four-quadrant chorionic villous tissue sampling sites, ~4 cm away from the umbilical cord insertion. The order of the four biopsies is clockwise starting at the main blood vessel. The maternally sided biopsy is taken at the basal side of the placenta at an equivalent position of biopsy 1. The basal plate is known to contain both cells of fetal (e.g., villous-lining syncytiotrophoblast) and maternal (e.g., polygonal decidual cells) origin, as also shown in Fig. 1. In our paper, and in general our birth cohort, we follow generally accepted methods for biopsy sampling and associated terminology^1–5^.

**Fig. 1:**
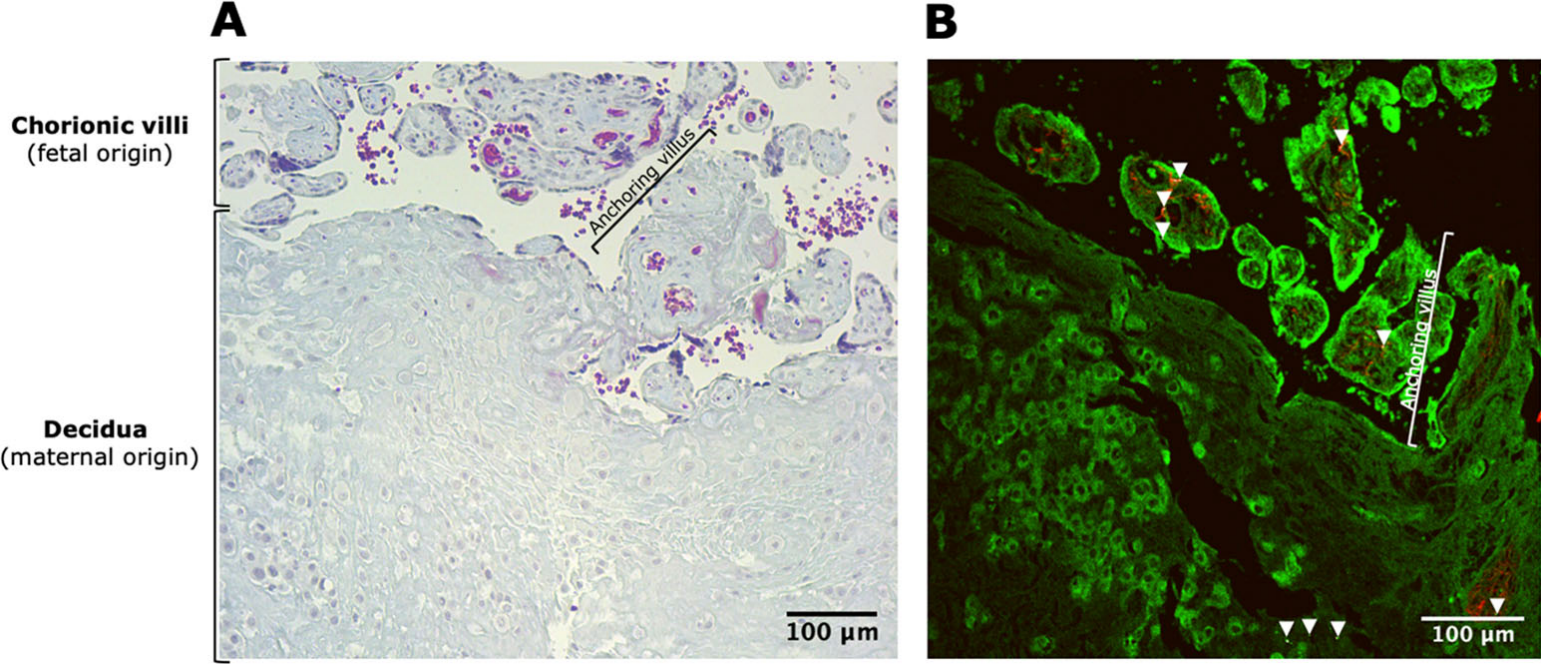
The term decidual basal plate. **A** Image from a histological section of a maternally sided placental biopsy stained with Masson’s trichrome. The maternally sided biopsies comprise decidual cells (maternal origin) as well as invaded extravillous trophoblasts and trabecular chorionic villi (both of fetal origin); those biopsies are thus composed of both maternal and fetal tissues. **B** Evidence of carbon particles in the maternally sided biopsy of the human placenta, as analyzed in the published manuscript. White-light generation originating from the carbon particles (white and further indicated using white arrowheads) under femtosecond pulsed laser illumination (excitation 810 nm) is observed in the placental tissue (two-photon excited autofluorescence from placental and red blood cells in green and second harmonic generation from collagen in red).

## Cellular composition of the placenta

Trophoblast cells constitute the placenta’s key cellular layer, which serves as a physical barrier to protect the fetus. In this regard, preferential uptake and accumulation of nanosized particles (e.g., polystyrene^7^ and gold^8^ nanoparticles) in the syncytiotrophoblast layer have been previously described. Our published images not only show the presence of carbon particles in the syncytiotrophoblast but also in the stroma of the villous placental tissue indicating that few particles can bypass the outermost cellular layer. Our paper sought to investigate whether combustion-derived particles are present in placental tissue and whether the placental black carbon load is related to ambient residential black carbon exposure during gestation. Overview images rather than detailed high resolution and high magnification images (using, for example, electron microscopy) were collected to analyze sufficiently large tissue areas for the presence of carbon particles and shown as such as being representative for the placental black carbon load as used in our performed quantitative analysis.

**Fig. 2:**
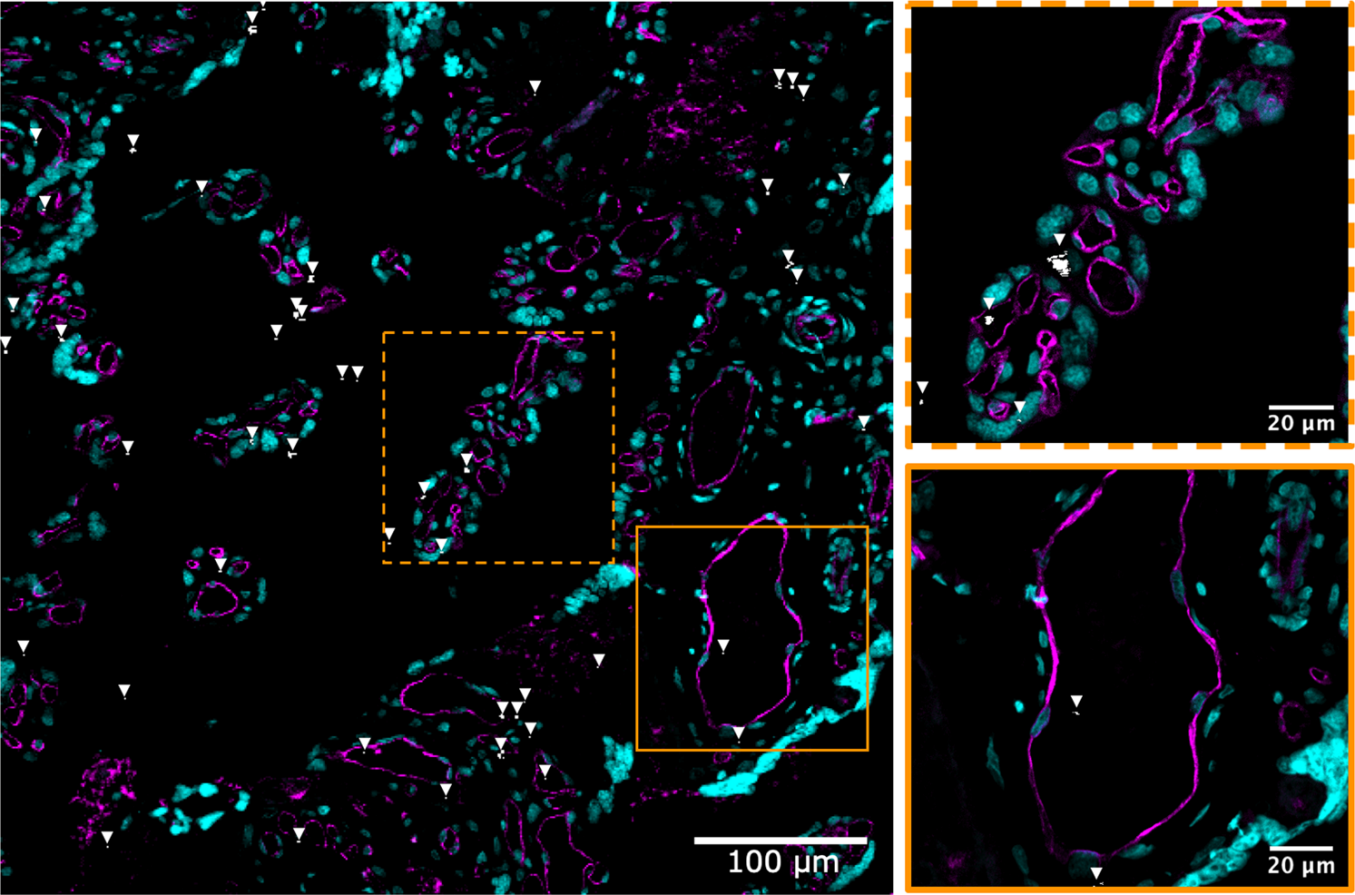
Confocal images of placental tissue sections indicating the presence of carbon particles inside fetal microvessels. Fetal capillaries were visualized using anti-CD31 antibodies directed against endothelial cells (magenta), and Syto 61 Red fluorescent nucleic acid stain (cyan) was used as a counterstain. The carbon particles (white and further indicated using white arrowheads) were imaged under femtosecond pulsed laser illumination (excitation 810 nm). Stainings were performed in triplicate and presented images are representative for samples collected from two placentas.

According to Holder et al., to demonstrate particle transfer across the placental barrier to the fetus, it needs to be established that particles (i) have crossed the syncytiotrophoblast and particles are present within the villous stroma and/or (ii) are localized in the endothelium of fetal blood vessels substantiated by co-localization of cell lineage-specific markers. First of all, carbon particles can be identified within the villous stroma of the placenta as already shown in the published images (Fig. 2, original manuscript). Second, we now show the transfer of air pollution-derived particles towards the fetal circulation by identifying black carbon particles in the central core of the chorionic villous tissue, more specifically inside the fetal capillaries, as visualized in Fig. 2. Also, our findings are further supported by a recent study by Liu et al. describing the uptake of inhaled air pollution-derived particles by macrophage-enriched placental cells isolated from healthy post-term placentae suggesting possible fetal transfer of such pollution-sourced nanoparticles^9^.

In summary, we unequivocally confirm our main conclusion that black carbon particles from environmental exposure reach the fetal side of the human placenta. However, we agree with Holder and colleagues that our findings, although in line with recent independent observations by other colleagues (e.g., presence of air pollution particles in placental tissue cells observed by Liu et al.^9^), require further research to understand direct fetal exposure more precisely. In this regard, we are currently studying the presence of particles in umbilical cord blood and its connection with the placental black carbon load. To use the words of Holder et al., “only by sufficiently rigorous scientific and technical approach can we reliably advance understanding of the environmental determinants of pregnancy outcome and offspring health”. By demonstrating, for the first time ever, the presence of black carbon particles in the human placenta, we contributed reliably to the understanding of possible particle translocation towards the fetus and insights into preventable adverse health effects that could potentially harm the next generation.

## Methods

Masson’s Trichrome was performed to assess the macroscopic structure of the placenta. The stained sections were visualized using a light microscope (Nikon SMZ800, Nikon Corporation, Japan) and acquired using a digital camera (Nikon Cs-Ri2, Nikon Corporation, Japan). Cellular distribution of black carbon particles in placental tissue sections was evaluated by staining endothelial cells. This experiment was performed in triplicate and representative images are shown. For marker expression analysis, 4-µm-thick placental paraffin sections were deparaffinized in xylene and rehydrated in a graded ethanol series and PBS. Next, the sections were submerged in a citrate buffer solution (10 mM, pH 6.0) for 40 min at 97 °C for antigen retrieval. Endogenous peroxidase activity was quenched by immersing the sections in 0.3% H_2_O_2_ in PBS for 10 min. After blocking non-specific binding sites with protein block (X0909, Agilent Dako, USA) for 60 min, tissue sections were probed with mouse monoclonal antibody against human CD31 (1:100, M0823, Agilent, USA) overnight at 4 °C. After washing, the tissue sections were incubated with an Alexa Fluor® 555 conjugated goat-anti mouse secondary antibody (1:500, A2122, Invitrogen, USA). The antibody was diluted in 10% protein block/PBS. SYTOTM 61 Red (1:1000, S11343, Invitrogen, USA) was used as a nuclear counterstain. Images with a 512 × 512-pixel resolution of stained tissue sections were collected at room temperature using a Zeiss LSM 880 (Carl Zeiss, Germany) and a Plan-Apochromat ×20/0.8 (Carl Zeiss, Germany) objective at a 4.1 µs pixel dwell time. The Alexa Fluor^®^ 555-labeled endothelial cells and SYTO^TM^ 61 Red-labeled nuclei were excited by using a 0.64 mW 543 He–Ne laser and a 5 mW 633 He–Ne laser, respectively. Band-pass filters 490–600 and 650–750 nm were used for filtering the emission signal from the labeled placental cells and nuclei, respectively. A femtosecond pulsed laser (810 nm, 150 fs, 80 MHz, MaiTai DeepSee, Spectra-Physics, USA) tuned to a central wavelength of 810 nm was used to excite the black carbon particles. Two-photon induced white-light emission of the carbon particles was acquired in the non-descanned mode after spectral separation and emission filtering using 400–410 and 450–650 nm band-pass filters. Thresholded and overlapping pixels in the narrow second harmonic generation channel (405/10 nm) and the broader two-photon excited autofluorescence channel (550/200 nm) were confirmed to be black carbon particles. The images were acquired by ZEN Black 2.0 software (Carl Zeiss, Germany).

### Reporting summary

Further information on research design is available in the Nature Research Reporting Summary linked to this article.

## Supplementary information

Reporting Summary

## Data Availability

Data sharing not applicable to this article as no datasets were generated or analyzed during the current study.
